# Long-Term Outcomes of Mitral Valve Repair Versus Replacement in Patients with Ischemic Mitral Regurgitation: A Retrospective Propensity-Matched Analysis

**DOI:** 10.3390/jcdd12040109

**Published:** 2025-03-22

**Authors:** Ismail M. Elnagar, Rawan Alghamdi, Murtadha H. Alawami, Ahmad Alshammari, Abdulmalik A. Almedimigh, Monirah A. Albabtain, Alaa AlGhamdi, Huda H. Ismail, Mostafa A. Shalaby, Khaled A. Alotaibi, Amr A. Arafat

**Affiliations:** 1 Adult Cardiac Surgery Department, Prince Sultan Cardiac Center, Riyadh 12233, Saudi Arabia; ismailelnaggar@kasralainy.edu.eg (I.M.E.); rawan3575@gmail.com (R.A.); mhalawami@pscc.med.sa (M.H.A.); asalshammari@pscc.med.sa (A.A.); aalmedimigh@gmail.com (A.A.A.); hismail@pscc.med.sa (H.H.I.); dr_mostafa_alaa@hotmail.com (M.A.S.); kaalotaibi@pscc.med.sa (K.A.A.); 2 Cardiothoracic Surgery Department, Cairo University, Cairo 11562, Egypt; 3 Cardiac Research Department, Prince Sultan Cardiac Center, Riyadh 12233, Saudi Arabia; muneera_2004@yahoo.com; 4 Health Research Center, Ministry of Defense Healthcare Services, Riyadh 12426, Saudi Arabia; alaajghamdi@gmail.com; 5 Cardiothoracic Surgery Department, Tanta University, Tanta 31111, Egypt

**Keywords:** ischemic mitral regurgitation, mitral valve repair, mitral valve replacement, survival, reintervention, left ventricular ejection fraction, left ventricular dimensions

## Abstract

Background: The optimal surgical management of ischemic mitral regurgitation (IMR)—mitral valve repair (MVr) versus mitral valve replacement (MVR)—remains controversial, with limited evidence on long-term outcomes. This study aimed to compare the outcomes of MVr and MVR in patients with IMR, focusing on survival and recurrence of mitral regurgitation. Additionally, survival was compared based on preoperative characteristics. Methods: A retrospective cohort analysis was conducted at a tertiary referral center and included 759 patients who underwent surgery for IMR between 2009 and 2021. Propensity score matching identified 140 matched pairs. The outcomes assessed included hospital mortality, long-term survival, recurrence of mitral regurgitation, mitral valve reintervention rates, and echocardiographic changes over time. Results: In the matched cohort, no significant differences were observed in hospital mortality (10% for MVr vs. 10.7% for MVR, *p* > 0.99) or long-term survival (*p* = 0.534). However, MVr was associated with a higher rate of recurrent moderate or higher mitral regurgitation (29.04% vs. 10.37%, *p* < 0.001) compared to MVR. The mitral valve reintervention rates did not differ significantly between the groups. Echocardiographic follow-up revealed significant improvements in left ventricular function and dimensions, with no significant differences between the groups. A subgroup analysis revealed no difference in survival according to the age, gender, ejection fraction, EuroSCORE category, or right ventricular function between the MVr and MVR patients. Conclusions: MVr and MVR for IMR yielded comparable survival rates, but MVr was associated with a higher risk of recurrent MR. The efficacy of both surgical approaches across diverse patient populations was comparable, reinforcing the need for individualized decision-making based on other clinical and anatomical considerations.

## 1. Introduction

Ischemic mitral regurgitation (IMR) is a complex and multifaceted complication of coronary artery disease that represents a ventricular rather than a valve disease [1,2,3]. It occurs in approximately 20% of patients following myocardial infarction and is more common in patients with associated heart failure [4,5]. IMR presents significant clinical challenges due to its high morbidity and mortality. Because of its complex mechanism, the management of IMR remains a controversial topic in cardiac surgery, and few recommendations in the recent American College of Cardiology/American Heart Association (ACC/AHA) guidelines are based on A level evidence, and no recommendations in the European Society of Cardiology (ESC) guidelines are a class 1 recommendation with level A evidence [6,7,8].

In patients undergoing surgical treatment for IMR, the decision between mitral valve repair (MVr) and mitral valve replacement (MVR) remains a complex and debated topic in cardiac surgery [9]. Several studies and meta-analyses have reported comparable early outcomes between MVr and MVR [10,11]. In a meta-analysis by Formica and associates, early mortality was greater in patients who underwent MVR; however, survival beyond two years was comparable between MVr and MVR [12]. Moreover, the durability of MVr in IMR is still controversial [10,13]. Despite existing research, unanswered questions remain regarding the long-term outcomes of surgical treatments for IMR. The ongoing debate centers on whether mitral valve repair or replacement offers superior benefits in terms of survival, functional status, and recurrence of mitral regurgitation. Furthermore, the optimal criteria for selecting patients for MVr versus MVR in IMR are not well defined, and there is no consensus on which patients benefit most from each approach. Therefore, the objectives of this study were to compare the outcomes of MVr and MVR in patients with IMR, with a focus on survival rates, postoperative complications, recurrence of mitral regurgitation, and long-term echocardiographic changes. Additionally, this study aimed to compare survival according to preoperative conditions such as age, gender, ejection fraction, right ventricular dysfunction, and EuroSCORE II.

## 2. Methods

### 2.1. Study Design

This study is a retrospective cohort analysis conducted at the Prince Sultan Cardiac Center in Riyadh, Saudi Arabia. Patients who underwent surgery for IMR from 2009 to 2021 were included. The study was approved by the local institutional review board (number: R20008). The need for patient consent was waived because the study was a retrospective analysis.

### 2.2. Patients

The study included patients diagnosed with IMR who underwent surgical intervention. A total of 759 patients were included, with 565 (74.4%) undergoing MVr and 194 (25.6%) undergoing MVR. Propensity score matching was employed, resulting in 280 matched patients (140 in each group). Patients were assigned to each group according to the surgeon’s preference; however, those with a coaptation depth of ≤10 mm generally underwent MVR. Mitral valve surgery was performed for moderate or higher degrees of regurgitation (Grade III or IV). In patients with Grade II mitral regurgitation, correction was performed in the case of a low ejection fraction (≤35%) or association with left ventricular dilatation (>110 mL/m^2^ end-diastolic volume).

### 2.3. Study Variables

Data were collected from the prospectively maintained Adult Cardiac Surgery Database. The study examined a range of preoperative, intraoperative, and postoperative variables, including demographic data (age and gender), comorbidities (diabetes mellitus, chronic obstructive pulmonary disease (COPD), myocardial infarction (MI), and atrial fibrillation), functional status (New York Heart Association (NYHA) class and Canadian Cardiovascular Society (CCS) class), echocardiographic parameters (ejection fraction, end-diastolic (LVEDD), end-systolic (LVESD) diameters, and right ventricular dysfunction and dilatation), and surgical data (concomitant coronary artery bypass grafting (CABG), tricuspid valve surgery, and cross-clamp and cardiopulmonary bypass (CPB) times). The variables were collected according to EuroSCORE II, which was used for risk stratification [14].

### 2.4. Study Outcomes

The study outcomes were hospital outcomes (mortality, stroke, and dialysis) and long-term outcomes (survival, mitral valve reintervention, recurrent moderate or higher mitral regurgitation, heart failure rehospitalization, functional status, and stroke).

### 2.5. Surgical Techniques

All patients in the study underwent surgical intervention via median sternotomy. The aorta, and superior and inferior vena cava were cannulated. The mitral valve was approached via a trans-septal incision. For patients undergoing MVR, the choice between tissue and mechanical valves was influenced by factors such as patient age, comorbidities, and the need for anticoagulation therapy. MVR was performed with preservation of all valve components and only a small elliptical portion of the anterior leaflet was excised. In the MVr group, all the patients had annuloplasty and the rings were downsized for the annular diameter. Limited leaflet resection was performed and chordal preservation was meticulously performed in 94.5% of the patients in both groups to maintain left ventricular function and geometry. Patch augmentation of the mitral valve was required in a small subset of patients (*n* = 5). In the case of residual mitral regurgitation of a moderate degree or higher after repair, MVR was performed.

### 2.6. Statistical Methods

The statistical analysis was performed using Stata 18 software (Stata Corp, College Station, TX, USA). Descriptive statistics are presented as the means ± standard deviations or medians with interquartile ranges (IQRs) for continuous variables, depending on their distribution, and as frequencies with percentages for categorical variables. Inferential statistics were used to compare groups based on the type of intervention, with a *p* value of <0.05 considered statistically significant. Comparisons of continuous data were performed via t tests or Mann–Whitney tests, and chi–squared tests or Fisher’s exact tests were used for categorical variables. Propensity score matching with 1:1 nearest neighbor matching was performed. The variables included in the propensity score model were selected based on their potential to influence the outcomes and the choice of treatment. These factors included age, EuroSCORE II, echocardiographic parameters, and concomitant surgical procedures. These variables were chosen because they are known to influence both the decision to perform MVr versus MVR and the outcomes of interest. The Caliber coefficient was 0.02, and the accepted percentage of bias correction was 20%. Supplementary Figure S1 shows the distribution of propensity scores between groups. The standardized mean difference was reported for the baseline data post-match. Comparisons of the outcomes were performed using the signed rank test for continuous data or the McNamar test for categorical data. The Kaplan–Meier method was used to plot the time-to-event data, and the log-rank test was used for comparisons before matching and Cox regression was used after matching. Subgroup analyses were conducted on the matched cohort to explore the impact of factors such as age, sex, ejection fraction, EuroSCORE II, and right ventricular dysfunction on survival outcomes via logistic regression analysis. Echocardiographic data were compared between groups via random effects, and the interactions between the groups and time were reported.

## 3. Results

### 3.1. Baseline Demographics and Clinical Characteristics

In the unmatched cohort, the mean age was similar between the groups (*p* = 0.388). Male patients were predominant in both groups but significantly more frequent in the MVr group (75.4% vs. 65.46%, *p* = 0.007). In the matched cohort, the age and gender were balanced. Comorbidities, including diabetes mellitus, were prevalent in both the unmatched (75.04% for repair vs. 68.75% for replacement, *p* = 0.088) and matched cohorts (72.14% vs. 70%, SMD = 0.05). Other comorbidities, such as COPD, atrial fibrillation, and dialysis, were not significantly different between the groups in either cohort. The functional classes (NYHA class III/IV and CCS class III/IV) were comparable between the groups in both cohorts. There was no difference in creatinine clearance between the groups; however, the EuroSCORE II was significantly greater in patients who underwent MVR with a preoperative intra-aortic balloon pump (IABP), and emergency surgery was required more frequently in patients who underwent MVR.

The echocardiographic data revealed significant differences in preoperative values in the unmatched cohort, with a larger LVEDD (57.22 ± 8.54 mm vs. 55.12 ± 6.86 mm, *p* < 0.001) and LVESD (43.65 ± 10.16 mm vs. 41.79 ± 7.96 mm, *p* = 0.01) in the MVR group. Right ventricular dysfunction and dilatation were more prevalent in the MVR group. These differences did not appear in the matched cohort (Table 1).

### 3.2. Intraoperative Characteristics

Concomitant CABG was nearly universal in both the unmatched (97.94% vs. 98.41%, *p* = 0.664) and matched cohorts (99.29% vs. 98.57%, SMD = 0.07). Tricuspid valve repair was more commonly performed concomitantly with MVR in the unmatched cohort (Table 1). MVR had longer cross-clamp times in the unmatched cohort (median 122 min [IQR: 104–142] vs. 111 min [IQR: 92–130], *p* < 0.001) and longer CPB times (median 155 min [IQR: 133–186] vs. 140 min [IQR: 120–167], *p* < 0.001). These differences were also present in the matched cohort (Table 2).

For patients undergoing MVR, tissue valves were predominantly used (83%), whereas mechanical valves were implanted in the remaining 17% of the patients. For MVr, the most commonly used annuloplasty ring was the SMB50 (Sovering MiniBand, SMB50, Sorin, Saluggia, Italy), which was deployed in 376 patients (66.5%).

### 3.3. Postoperative Outcomes

In the unmatched cohort, MVR was associated with higher rates of new-onset atrial fibrillation (*p* = 0.01) and extracorporeal membrane oxygenation (ECMO) use (*p* = 0.003). However, these differences were not significant in the matched cohort (AF: *p* > 0.99; ECMO: *p* = 0.219). Hospital mortality was higher in the MVR group in the unmatched cohort (12.89% vs. 6.73%, *p* = 0.007) but not in the matched cohort (10.7% vs. 10%, *p* > 0.99) (Table 2).

### 3.4. Long-Term Outcomes

**Figure 1:**
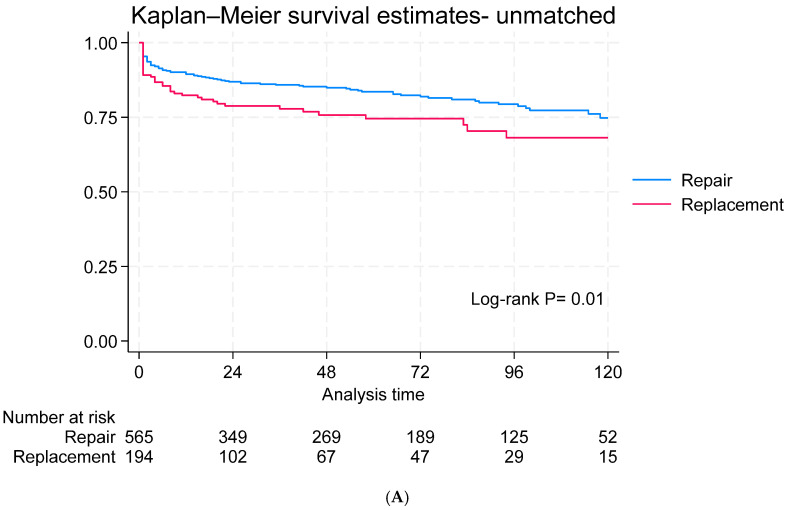

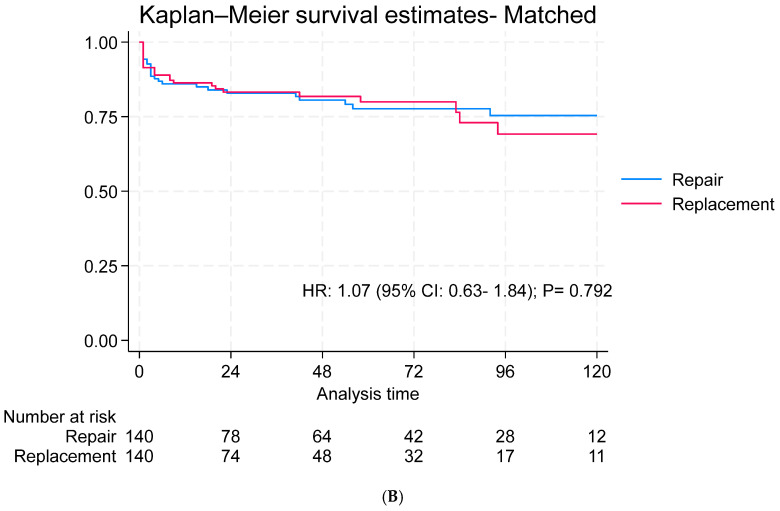
Kaplan–Meier survival in the unmatched (**A**) and matched groups (**B**).

**Figure 2:**
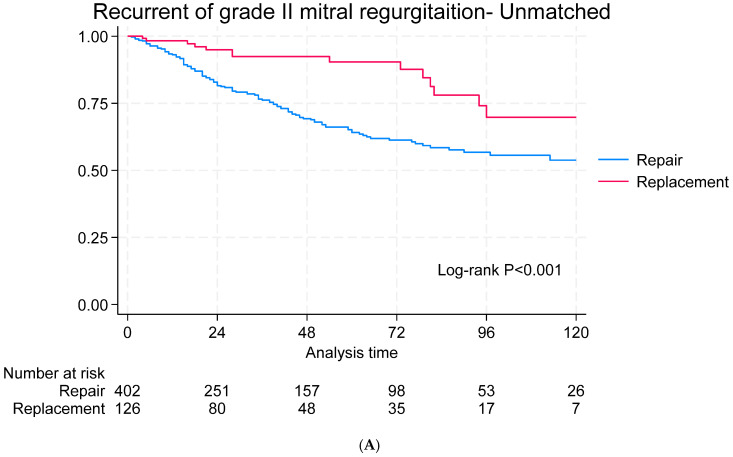

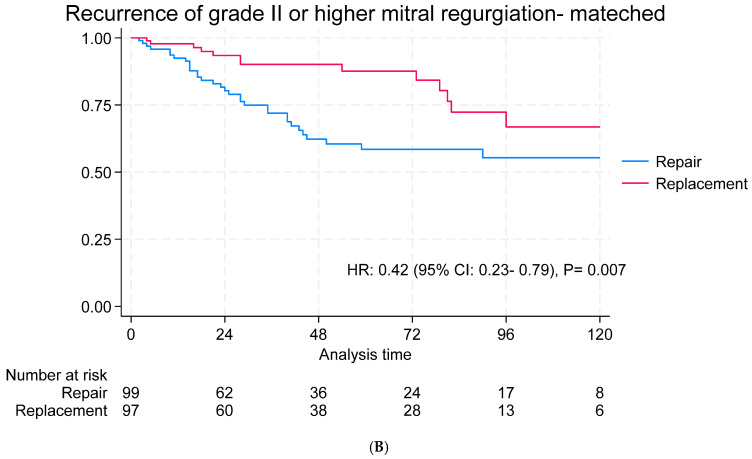
Recurrence of Grade II or higher mitral regurgitation in the unmatched (**A**) and matched (**B**) groups.

**Figure 3:**
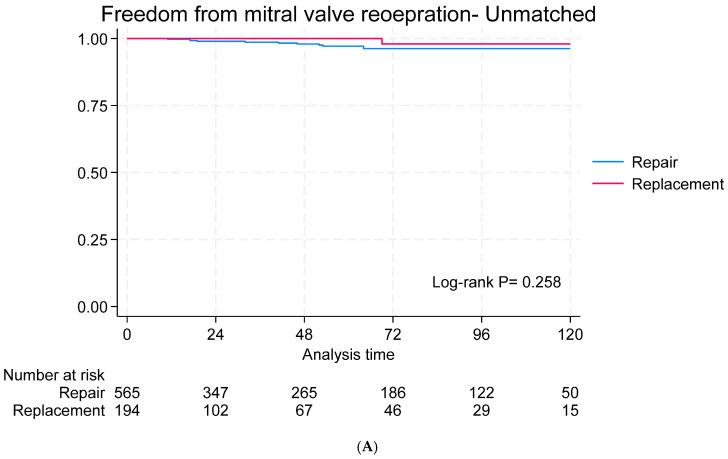

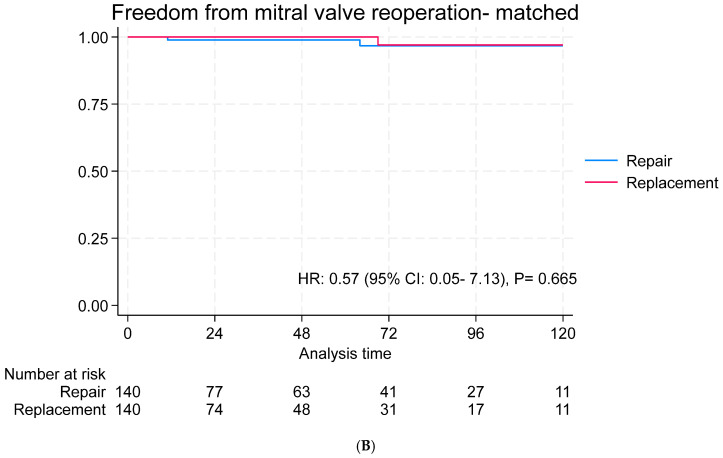
Freedom from mitral valve reoperation in the unmatched (**A**) and unmatched (**B**) groups.

The median follow-up period was 38 months (IQR: 6–85). The survival rates at 1, 5, and 10 years were 89%, 83%, and 74%, respectively, for the MVr group and 82%, 74%, and 67%, respectively, for the MVR group (*p* < 0.001). In the matched cohort, no difference in survival was reported (Figure 1A,B). Recurrent mitral regurgitation was observed in 29.04% of the repair group and 10.37% of the replacement group and it was significantly higher in the MVr repair group in both the unmatched (*p* < 0.001) and matched cohorts (*p* = 0.007) (Figure 2A,B). Twelve patients underwent mitral valve surgery: eleven in the MVr group (three with stenosis and eight with regurgitation) and one in the MVR group (bioprosthetic valve degeneration). Freedom from mitral valve surgery did not differ significantly between the matched and unmatched cohorts (Figure 3A,B). There was no difference in heart failure rehospitalization (Supplementary Figure S2A,B), stroke (Supplementary Figure S3A,B), or freedom from NYHA III/IV (Supplementary Figure S4A,B) between the matched and unmatched groups.

### 3.5. Subgroup Analysis for Survival

A detailed subgroup analysis was conducted to compare survival outcomes between MVr and MVR across various patient demographics and clinical parameters. The analysis revealed no significant differences in survival between MVr and MVR in several key subgroups, including patients younger or older than 60 years, males and females, those with a EuroSCORE II greater than or less than 8, and patients with an ejection fraction greater or less than 40%. Additionally, no significant survival differences were observed in terms of right ventricular function (Table 3).

### 3.6. Echocardiographic Follow-Up

A total of 1680 echocardiographic studies were available. The EF improved significantly over a 7-year follow-up (β: 0.04 (0.02–0.05), *p* < 0.001), with no significant difference between the unmatched (β: −0.02 (95% CI: −0.06–0.01), *p* = 0.185) and matched (β: −0.02 (95% CI: −0.07–0.04), *p* = 0.554) cohorts. The LVEDD was significantly reduced (β: −0.5 (95% CI: −0.07–−0.04), *p* < 0.001) but the reduction was not significantly different between the unmatched (β: −0.005 (95% CI: −0.03–0.02), *p* = 0.744) and matched (β: −0.01 (95% CI: −0.05–0.03), *p* = 0.655) cohorts. Similarly, the LVESD decreased during follow-up (β: −0.04 (95% CI: −0.05–−0.02), *p* < 0.001), and there was no difference between the unmatched (β: 0.03 (95% CI: −0.01–0.06), *p* = 0.113) and matched (β: 0.02 (95% CI: −0.02–0.07), *p* = 0.354) cohorts.

## 4. Discussion

This retrospective cohort study compared the outcomes of MVr and MVR in 759 patients with IMR. Using propensity score matching, 280 patients were analyzed for hospital mortality, long-term survival, recurrence of mitral regurgitation, and reintervention rates. The results revealed no significant difference in hospital mortality or long-term survival between the two groups. However, MVr was associated with a significantly higher rate of recurrent mitral regurgitation compared to MVR. Echocardiographic improvements in left ventricular function and dimensions were observed in both groups, with no significant differences. The subgroup analysis suggested comparable outcomes between both approaches in different clinical parameters. We also reported higher cardiopulmonary bypass and ischemic times in patients who had MVR, which could be attributed to the conversion from repair to replacement in some patients.

### 4.1. Hospital Outcomes

The comparison of hospital outcomes between MVr and MVR in patients with IMR highlights distinct advantages and limitations for each surgical approach. In a landmark clinical randomized trial conducted by Acker and colleagues, no significant differences were observed between MVr and MVR in terms of 30-day and 1-year outcomes; however, the replacement group experienced higher rates of rehospitalization [11]. Similarly, a meta-analysis found that patients undergoing MVr had a non-significantly better survival rate compared to those undergoing MVR, though the difference did not reach statistical significance [10]. Another meta-analysis by Vassileva and colleagues reported a higher likelihood of short-term mortality in patients who underwent MVR, suggesting potential risks associated with replacement procedures [15]. The improved outcomes observed in MVr patients could be attributed to the preservation of left ventricular geometry and function, which is often compromised in IMR due to the underlying ischemic pathology [16]. In contrast to our results, Dufendach and associates reported higher operative mortality and infection rates, prolonged ventilation, and renal failure in patients undergoing MVR compared to MVr, which further complicates the decision-making process [17]. These findings have contributed to the growing preference for MVr as the primary surgical option for managing IMR. However, it is important to note that these meta-analyses primarily included observational studies, which may introduce bias, and the clinical trial by Acker and colleagues demonstrated comparable outcomes between the two approaches, which regained equivalence between both approaches [11]. A meta-analysis published by Takagi and associates that included randomized clinical trials and adjusted observational studies reported comparable early and late outcomes between MVr and MVR [18].

Our study reported no differences in hospital mortality and adverse events between the two groups. This finding suggests that, in this specific subgroup of patients with IMR, the outcomes may be more strongly influenced by preoperative clinical conditions (e.g., ventricular function and comorbidities) than by the type of surgical intervention. This observation aligns with the understanding that IMR is primarily a ventricular disease rather than a valvular disease, as emphasized in the literature [1,2,3]. The underlying ischemic cardiomyopathy and ventricular remodeling play a critical role in determining the outcomes, regardless of whether MVr or MVR is performed. The current European guidelines emphasize the importance of Optimal Medical Therapy (OMT), including cardiac resynchronization therapy (CRT) when indicated, as the cornerstone of management for patients with IMR [7]. OMT has been shown to improve symptoms, reduce hospitalizations, and enhance the quality of life in these patients. The guidelines primarily recommend valve surgery for patients who require concomitant CABG or other cardiac surgeries, as reflected in our study population, where nearly all patients underwent concomitant CABG. This underscores the importance of addressing the underlying ischemic pathology in addition to managing the valvular dysfunction.

### 4.2. Long-Term Outcomes

The long-term outcomes following MVr versus MVR remain a topic of ongoing debate [19]. Thourani and colleagues reported higher 10-year survival rates in patients younger than 60 years who underwent MVr, whereas no significant survival difference was detected between the two approaches in patients older than 60 years [20]. In the Polish National Registry, MVr was associated with better long-term survival compared to MVR [21]. In a meta-analysis of 12 studies, Rao and colleagues reported improved long-term outcomes following MVr; however, the analysis included heterogeneous studies, leading to considerable uncertainty in the conclusions [22]. Another study highlighted that the survival difference between MVr and MVR diminished when concomitant coronary artery bypass grafting was performed [20]. In our study, we accounted for concomitant procedures through propensity score matching, which may have contributed to the comparable survival rates observed between MVr and MVR. Virk and associates conducted a comprehensive meta-analysis of 22 observational studies and 1 clinical trial comparing MVr and MVR for IMR [23]. Their findings indicated reduced mortality rates following MVr, particularly in studies reporting outcomes beyond three years. However, the recurrence of moderate or higher-grade mitral regurgitation was significantly higher in the repair group, a finding consistently supported by other meta-analyses [10,23]. Kron and coworkers, in a subgroup analysis from the Cardiothoracic Surgical Trials Network, reported that 76 out of 116 patients who underwent MVr developed a moderate or higher degree of mitral regurgitation during follow-up [24]. The most common mechanism of recurrent mitral regurgitation was mitral valve leaflet tethering, which was strongly associated with basal aneurysm or dyskinesia [24]. One of the notable findings of our study is the higher recurrence of mitral regurgitation in the repair group, which was not accompanied by a corresponding increase in rehospitalization for heart failure or reoperation rates. This observation can be attributed to several key factors. First, the recurrence of mitral regurgitation in our study was defined as a moderate or higher degree of regurgitation. However, reoperation is typically reserved for cases of severe, symptomatic MR. Second, the progression of recurrent MR in these patients may have been gradual, allowing for physiological adaptation to the increasing regurgitant volumes over time. Third, as highlighted in recent guidelines [7], recurrent mitral regurgitation can often be effectively managed with OMT that can help control symptoms and prevent heart failure exacerbations, even in the presence of moderate MR. Sweeney and colleagues reported similar early and late mortality and degree of mitral regurgitation between repair and replacement at the 2-year follow-up [25]. These findings underscore the complexity of decision-making in IMR management, balancing the benefits of MVr in preserving ventricular function against the risk of recurrent regurgitation.

Few studies reported improvements in functional outcomes after repair compared to replacement, mainly because of the better preservation of left ventricular function [26]. In our series, we did not report differences between both approaches in the freedom from hospitalization due to heart failure and NYHA class III/IV. This is consistent with previous reports comparing repair and replacement in ischemic cardiomyopathy [11,27]. Both MVr and MVR improve the left ventricular end-systolic volume index (LVESVI) and reverse remodeling in ischemic cardiomyopathy patients. However, patients without recurrent MR after repair tend to show better improvement in LVESVI compared to those with recurrence [28]. The 2-year outcomes of the CTSN randomized trial reported no difference in ventricular remodeling between repair and replacement; however, repair was associated with a higher recurrence of regurgitation and increase in heart failure symptoms and admission [29]. In this study, we did not report differences between repair and replacement in terms of changes in left ventricular function and diameters. The comparable outcomes in our series could be attributed to the chordal preservation performed in most patients in the repair or replacement group, similar to the CTSN trial [28].

The findings suggest that while MVr and MVR offer comparable survival outcomes for IMR patients, MVr carries a higher risk of recurrent mitral regurgitation. This highlights the importance of individualized surgical decision-making. These results contribute to the ongoing debate on the optimal surgical approach for IMR and may inform future clinical guidelines. However, further research, particularly randomized controlled trials, is needed to confirm these findings and refine surgical strategies.

### 4.3. Limitations

This study has several limitations, including its retrospective design, which introduces potential biases and limits the ability to establish causal relationships. The use of propensity score matching, while helpful, cannot fully account for unmeasured confounders. The study was conducted at a single center, which may limit the generalizability of the findings to other populations or healthcare settings. Additionally, the follow-up period, while substantial, may not capture very long-term outcomes, particularly for younger patients. The reliance on surgeon preference for selecting MVr or MVR may have also introduced selection bias. Finally, the study did not explore the impact of newer surgical techniques or transcatheter interventions, which could influence future management strategies for IMR [30].

## 5. Conclusions

This study demonstrated that MVr and MVR for IMR yielded comparable survival rates, but MVr was associated with a higher risk of recurrent mitral regurgitation. The efficacy of both surgical approaches across diverse patient populations was comparable, reinforcing the need for individualized decision-making based on other clinical and anatomical considerations.

## Figures and Tables

**Table 1:** Preoperative and operative baseline demographics of patients receiving mitral valve repair vs. replacement for ischemic mitral regurgitation.

	Unmatched			Matched		
	Mitral Valve Repair (*n* = 565)	Mitral Valve Replacement (*n* = 194)	*p* Value	Mitral Valve Repair (*n* = 140)	Mitral Valve Replacement (*n* = 140)	SMD
Age (years)	62.46 ± 9.28	63.13 ± 9.58	0.388	63.28 ± 9.61	63.17 ± 9.24	0.01
Male	426 (75.40%)	127 (65.46%)	0.007	88 (62.86%)	86 (61.43%)	−0.03
BMI (kg/m^2^)	27 (25–31) (*n* = 563)	27 (24–31)	0.944	27 (24–31)	27 (24–31)	0.01
Smoker	59/443 (13.32%)	19/150 (12.67%)	0.838	12 (8.57%)	17 (12.14%)	−0.12
EuroSCORE II (%)	4.56 (2.57–7.58) (*n* = 535)	7.13 (3.8–12.18) (*n* = 191)	<0.001	6.1 (3.33–9.57)	5.89 (3.44–10.09)	0.02
NYHA III/IV	446/558 (79.93%)	161/192 (83.85%)	0.232	118 (84.29%)	118 (84.29%)	0
CCS III/IV	230/560 (41.07%)	65/192 (33.85%)	0.077	54 (38.57%)	43 (30.71%)	0.16
Diabetes mellitus	421/561 (75.04%)	132/192 (68.75%)	0.088	101 (72.14%)	98 (70%)	0.05
Dialysis	29/547 (5.30)	9/185 (4.86)	0.817	7 (5%)	6 (4.29%)	0.03
COPD	19/550 (3.45%)	5/185 (2.70%)	0.619	4 (2.86%)	4 (2.86%)	0
Previous MI	266/559 (47.58%)	87/189 (46.03%)	0.712	64 (45.71%)	54 (38.57%)	0.14
Atrial fibrillation	46/534 (8.61)	22/180 (12.22%)	0.154	17 (12.23%)	19 (13.87%)	−0.05
Implanted device	9/544 (1.65%)	5/179 (2.79%)	0.337	4 (2.96%)	3 (2.27%)	0.04
Previous cardiac surgery	11/557 (1.97)	8/193 (4.15)	0.098	4 (2.86%)	4 (2.86%)	0
Creatinine clearance (mL/min)	80 (56–102) (*n* = 543)	78 (58–95) (*n* = 189)	0.387	71 (52–95)	77 (58–95)	−0.05
Preop IABP	12/552 (2.17%)	13/187 (6.95%)	0.002	5 (3.60%)	7 (5%)	−0.07
Inotropes	74/550 (13.45%)	19/188 (10.11%)	0.232	17 (12.23%)	10 (7.14%)	0.17
Ventilation	60/550 (10.91%)	12/187 (6.42%)	0.074	11 (7.91%)	6 (4.29%)	0.15
Emergency	43 (7.61)	24 (12.37)	0.044	11 (7.86%)	12 (8.57%)	−0.03
Ejection fraction (%)	35 (30–45) (*n* = 556)	35 (25–45) (*n* = 194)	0.103	35 (30–45)	40 (28–48)	−0.12
LVEDD (mm)	55.12 ± 6.86 (*n* = 559)	57.22 ± 8.54 (*n* = 189)	<0.001	56 (52–60)	56 (52–61)	0
LVESD (mm)	41.79 ± 7.96 (*n* = 556)	43.65 ± 10.16 (*n* = 189)	0.010	42 (37–48)	43 (36–48)	0.06
RV hypofunction	38/558 (6.81%)	38/188 (20.21%)	<0.001	24 (17.14%)	24 (17.14%)	0
RV dilatation	28/564 (4.96%)	35/193 (18.13%)	<0.001	14 (10%)	22 (15.71%)	−0.17
Concomitant CABG	556 (98.41%)	190 (97.94%)	0.664	139 (99.29%)	138 (98.57%)	0.07
Tricuspid repair	171 (30.27%)	120 (61.86%)	<0.001	89 (63.57%)	86 (61.43%)	0.04

BMI: body mass index; CABG: coronary artery bypass grafting; CCS: Canadian Cardiovascular Society; COPD: chronic obstructive pulmonary disease; LVEDD: left ventricular end-diastolic diameter; LVESD: left ventricular end-systolic diameter; IABP: intra-aortic balloon pump; MI: myocardial infarction; NYHA: New York Heart Association; SMD: standardized mean difference. The data are presented as numbers (%), means (SDs), or medians (IQRs).

**Table 2:** Postoperative mitral valve repair vs. replacement for ischemic mitral regurgitation.

	Unmatched			Matched		
	Mitral Valve Repair (*n* = 565)	Mitral Valve Replacement (*n* = 194)	*p* Value	Mitral Valve Repair (*n* = 140)	Mitral Valve Replacement (*n* = 140)	*p* Value
Cross clamp (min)	111 (92–130) (*n* = 507)	122 (104–142) (*n* = 182)	<0.001	114 (94–130)	123 (105–143)	<0.001
CPB time (min)	140 (120–167) (*n* = 511)	155 (133–186) (*n* = 18)	<0.001	151 (125–172)	157 (134–187)	0.021
New-onset AF	67/491 (13.65%)	37/168 (22.02%)	0.010	21/121 (17.36%)	23/122 (18.85%)	>0.99
IABP	33/512 (6.45%)	29/177 (16.38%)	<0.001	8/123 (6.50%)	16/126 (12.70%)	0.151
ECMO	11/513 (2.14%)	12/177 (6.78%)	0.003	1/123 (0.81%)	5/126 (3.97%)	0.219
CRRT/Dialysis	40/509 (7.85%)	24/167 (14.37%)	0.013	15/121 (12.40%)	16/119 (13.45%)	0.832
Stroke	13/507 (2.56%)	11/168 (6.55%)	0.016	5/120 (4.17%)	10/120 (8.33%)	0.549
Open Sternum	18/505 (3.56%)	19/174 (10.92%)	<0.001	5/122 (4.10%)	8/128 (6.25%)	0.581
Hospital mortality	38 (6.73%)	25 (12.89%)	0.007	14 (10%)	15 (10.71%)	>0.99

AF: atrial fibrillation; CPB: cardiopulmonary bypass; ECMO: extracorporeal membrane oxygenation; CRRT: continuous renal replacement therapy; IABP: intra-aortic balloon pump. The data are presented as numbers (%), means (SDs), or medians (IQRs).

**Table 3:** Subgroup analysis of survival for mitral valve repair vs. replacement for ischemic mitral regurgitation.

	HR (95% CI)	*p*
Age		
Age < 60—Replacement vs. Repair	0.68 (0.20–2.28)	0.534
Age ≥ 60—Replacement vs. Repair	1.09 (0.59–2.01)	0.776
Gender		
Male—Replacement vs. Repair	0.93 (0.45–1.91)	0.845
Female—Replacement vs. Repair	1.29 (0.57–2.90)	0.536
Ejection fraction		
EF > 40—Replacement vs. Repair	1.72 (0.75–3.92)	0.202
EF ≤ 40—Replacement vs. Repair	0.74 (0.36–1.55)	0.427
EuroSCORE II		
EuroSCORE < 8—Replacement vs. Repair	1.25 (0.58–2.67)	0.564
EuroSCORE ≥ 8—Replacement vs. Repair	0.86 (0.40–1.84)	0.693
RV function		
Normal—Replacement vs. Repair	0.98 (0.54–1.78)	0.958
Dysfunction—Replacement vs. Repair	1.44 (0.59–3.56)	0.425

## Data Availability

Data sharing requires institutional approval according to the institutional regulations.

## Supplementary Materials

The following supporting information can be downloaded at: https://www.mdpi.com/article/10.3390/jcdd12040109/s1, Figure S1: mirror graph of the propensity score distribution. Figure S2: Freedom from heart failure rehospitalization. Figure S3: Freedom from stroke. Figure S4: Freedom from NYHA III/IV.

## References

[1] Grigioni F., Enriquez-Sarano M., Zehr K.J., Bailey K.R., Tajik A.J. (2001). Ischemic Mitral Regurgitation. Circulation.

[2] Estévez-Loureiro R., Lorusso R., Taramasso M., Torregrossa G., Kini A., Moreno P.R. (2024). Management of Severe Mitral Regurgitation in Patients with Acute Myocardial Infarction: JACC Focus Seminar 2/5. J. Am. Coll. Cardiol..

[3] Hadjadj S., Marsit O., Paradis J.M., Beaudoin J. (2021). Pathophysiology, Diagnosis, and New Therapeutic Approaches for Ischemic Mitral Regurgitation. Can. J. Cardiol..

[4] Chaput M., Handschumacher M.D., Tournoux F., Hua L., Guerrero J.L., Vlahakes G.J., Levine R.A. (2008). Mitral Leaflet Adaptation to Ventricular Remodeling. Circulation.

[5] Huang A.L., Dal-Bianco J.P., Levine R.A., Hung J.W. (2023). Secondary Mitral Regurgitation: Cardiac Remodeling, Diagnosis, and Management. Struct. Heart.

[6] Otto C.M., Nishimura R.A., Bonow R.O., Carabello B.A., Erwin J.P., Gentile F., Jneid H., Krieger E.V., Mack M., McLeod C. (2021). 2020 ACC/AHA Guideline for the Management of Patients With Valvular Heart Disease: Executive Summary: A Report of the American College of Cardiology/American Heart Association Joint Committee on Clinical Practice Guidelines. Circulation.

[7] Vahanian A., Beyersdorf F., Praz F., Milojevic M., Baldus S., Bauersachs J., Capodanno D., Conradi L., De Bonis M., De Paulis R. (2022). 2021 ESC/EACTS Guidelines for the management of valvular heart disease: Developed by the Task Force for the management of valvular heart disease of the European Society of Cardiology (ESC) and the European Association for Cardio-Thoracic Surgery (EACTS). Eur. Heart J..

[8] Nappi F., Singh S.S.A., Fiore A., Ellouze O. (2022). Insight from International Guidelines: Do We Have Satisfactory Recommendations for Secondary Mitral Regurgitation?. Rev. Cardiovasc. Med..

[9] Hu J., Lee A.P.W., Wei X., Cheng Z.Y., Ho A.M.H., Wan S. (2022). Update on surgical repair in functional mitral regurgitation. J. Card. Surg..

[10] Gamal M.A., El-Fiky M.M., Gamea M.M., Ali I. (2022). Mitral valve repair versus replacement in severe ischemic mitral regurgitation systematic review and meta-analysis. J. Card. Surg..

[11] Acker M.A., Parides M.K., Perrault L.P., Moskowitz A.J., Gelijns A.C., Voisine P., Smith P.K., Hung J.W., Blackstone E.H., Puskas J.D. (2014). Mitral-valve repair versus replacement for severe ischemic mitral regurgitation. N. Engl. J. Med..

[12] Formica F., Gallingani A., Tuttolomondo D., Hernandez-Vaquero D., D’Alessandro S., Singh G., Benassi F., Grassa G., Pattuzzi C., Maestri F. (2024). Long-term outcomes comparison of mitral valve repair or replacement for secondary mitral valve regurgitation. An updated systematic review and reconstructed time-to-event study-level meta-analysis. Curr. Probl. Cardiol..

[13] Luz F.M.-H., Amorim M.J. (2022). Ischemic mitral regurgitation—To repair or replace? looking beyond the valve. Port. J. Card. Thorac. Vasc. Surg..

[14] Nashef S.A.M., Roques F., Sharples L.D., Nilsson J., Smith C., Goldstone A.R., Lockowandt U. (2012). EuroSCORE II. Eur. J. Cardio-Thorac. Surg..

[15] Vassileva C.M., Boley T., Markwell S., Hazelrigg S. (2011). Meta-analysis of short-term and long-term survival following repair versus replacement for ischemic mitral regurgitation. Eur. J. Cardio-Thorac. Surg..

[16] Hannan E.L., Samadashvili Z., Smith C.R., Lahey S.J., Gold J.P., Jordan D., Sundt T.M., Girardi L., Ashraf M.H., Chikwe J. (2019). Mitral valve repair versus replacement for patients with preserved left ventricular function without heart failure symptoms. J. Thorac. Cardiovasc. Surg..

[17] Dufendach K., Aranda-Michel E., Sultan I., Gleason T.G., Navid F., Thoma F., Kilic A. (2020). Outcomes of mitral valve surgery for severe ischemic mitral regurgitation. J. Card. Surg..

[18] Takagi H., Umemoto T. (2016). Similar Survival After Repair vs Replacement for Ischemic Mitral Regurgitation. Semin. Thorac. Cardiovasc. Surg..

[19] Di Mauro M., Cargoni M., Liberi R., Lorusso R., Calafiore A.M. (2022). Mitral valve repair or replacement. How long is this feud to last?. J. Card. Surg..

[20] Thourani V.H., Weintraub W.S., Guyton R.A., Jones E.L., Williams W.H., Elkabbani S., Craver J.M. (2003). Outcomes and Long-Term Survival for Patients Undergoing Mitral Valve Repair Versus Replacement. Circulation.

[21] Deja M.A., Malinowski M., Widenka K., Stożyński N., Bartuś K., Kapelak B., Kuśmierczyk M., Hrapkowicz T., Suwalski P., Jasiński M. (2022). Repair or Replacement for Secondary Mitral Regurgitation: Results From Polish National Registry. Ann. Thorac. Surg..

[22] Rao C., Murphy M.O., Saso S., Pandis D., Grapsa J., Nihoyannopoulos P., Reeves B.C., Athanasiou T. (2011). Mitral Valve Repair or Replacement for Ischaemic Mitral Regurgitation: A Systematic Review. Heart Lung Circ..

[23] Virk S.A., Sriravindrarajah A., Dunn D., Liou K.P., Wolfenden H.D., Tan G.M.Y., Cao C. (2015). A meta-analysis of mitral valve repair versus replacement for ischemic mitral regurgitation. Ann. Cardiothorac. Surg..

[24] Kron I.L., Hung J., Overbey J.R., Bouchard D., Gelijns A.C., Moskowitz A.J., Voisine P., O’Gara P.T., Argenziano M., Michler R.E. (2015). Predicting recurrent mitral regurgitation after mitral valve repair for severe ischemic mitral regurgitation. J. Thorac. Cardiovasc. Surg..

[25] Sweeney J.C., Alotaibi A., Porter G.D., Avula D., Trivedi J.R., Slaughter M.S., Ganzel B.L., Pahwa S.V. (2024). Ischemic mitral regurgitation: To repair or replace? A single center experience. PLoS ONE.

[26] McNeely C.A., Vassileva C.M. (2014). Long-term Outcomes of Mitral Valve Repair Versus Replacement for Degenerative Disease: A Systematic Review. Curr. Cardiol. Rev..

[27] Arafat A.A., Alghamdi R., Alfonso J., Shalaby M.A.A., Alotaibi K., Pragliola C. (2023). Concomitant Mitral Valve Repair vs Replacement During Surgical Ventricular Restoration for Ischemic Cardiomyopathy. Angiology.

[28] LaPar D.J., Acker M., Gelijns A.C., Kron I.L. (2015). Repair or replace for severe ischemic mitral regurgitation: Prospective randomized multicenter data. Ann. Cardiothorac. Surg..

[29] Goldstein D.R., Moskowitz A.J., Gelijns A.C., Ailawadi G., Parides M.K., Perrault L.P., Hung J.W., Voisine P., Dagenais F., Gillinov A.M. (2016). Two-Year Outcomes of Surgical Treatment of Severe Ischemic Mitral Regurgitation. N. Engl. J. Med..

[30] Noly P.E., Pagani F.D., Obadia J.F., Bouchard D., Bolling S.F., Ailawadi G., Tang P.C. (2022). The role of surgery for secondary mitral regurgitation and heart failure in the era of transcatheter mitral valve therapies. Rev. Cardiovasc. Med..

