# Supplementary material for: Long-Term Outcomes of Mitral Valve Repair Versus Replacement in Patients with Ischemic Mitral Regurgitation: A Retrospective Propensity-Matched Analysis
Source: J Cardiovasc Dev Dis. 2025 Mar 22;12(4):109. doi: 10.3390/jcdd12040109 (PMC12027820; doi:10.3390/jcdd12040109)
Supplement: Supplementary file 1 [file jcdd-12-00109-s001.zip › jcdd-3549749-supplementary.pdf]

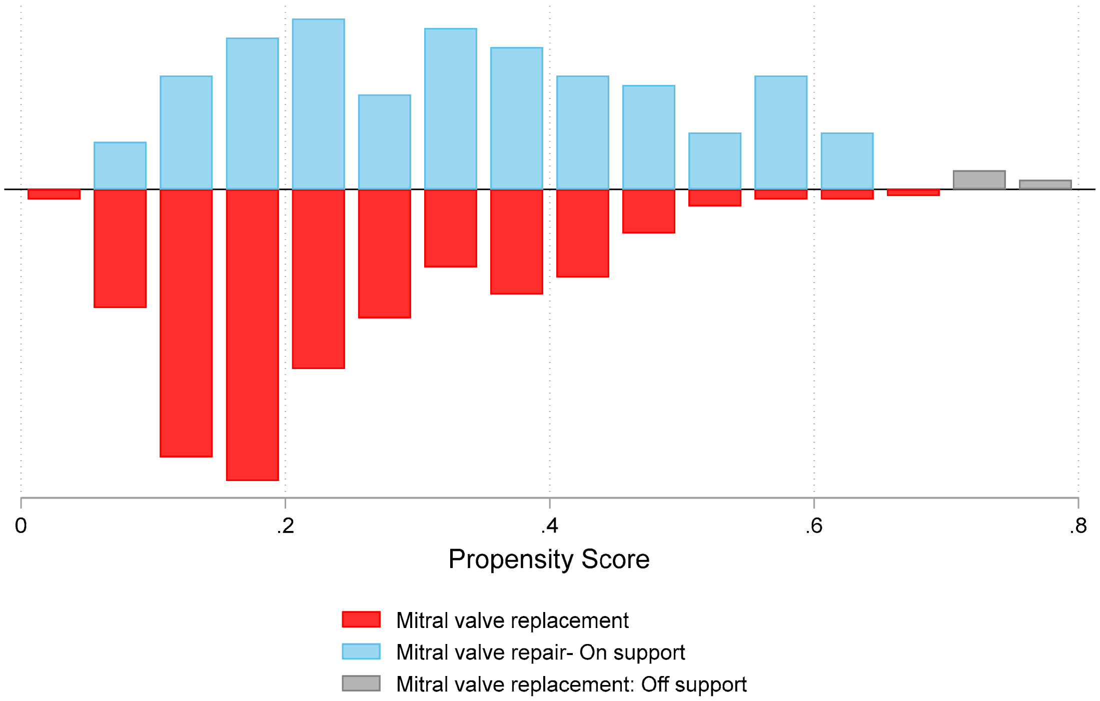

Figure S1: Mirror graph of the propensity score distribution between patients who had mitral valve replacement and repair. On-support refers to patients whose propensity scores fall within the common support region and were matched; however, off-support refers to those with propensity score outside the common support region.

A

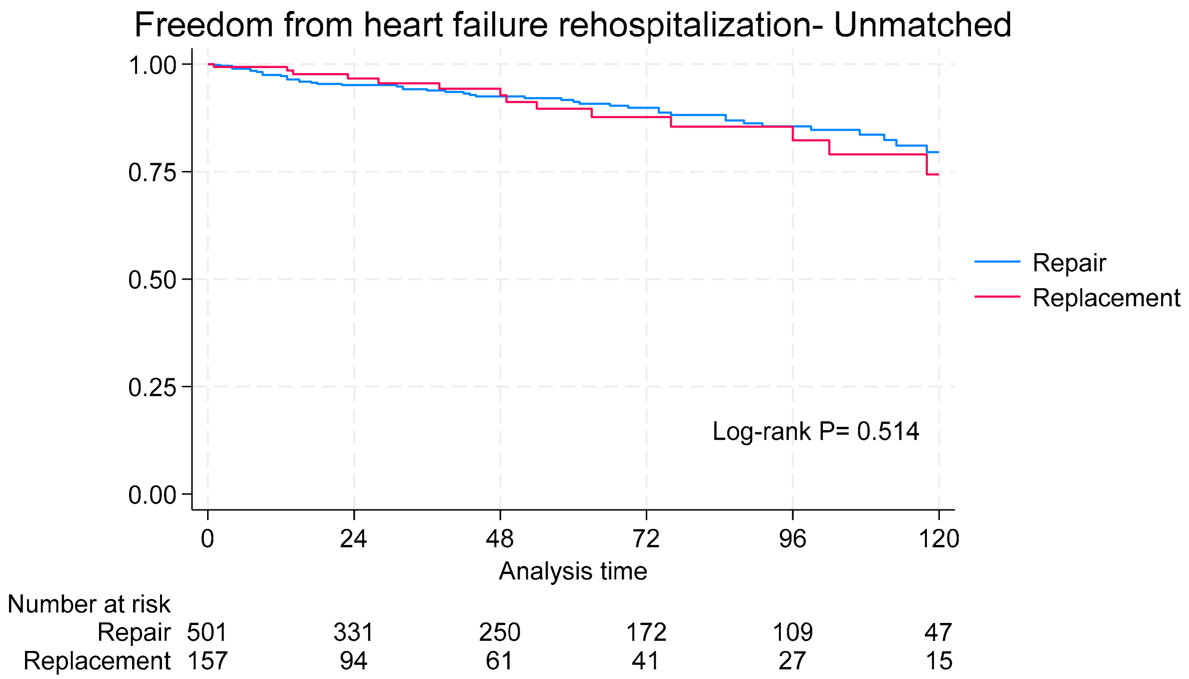

B

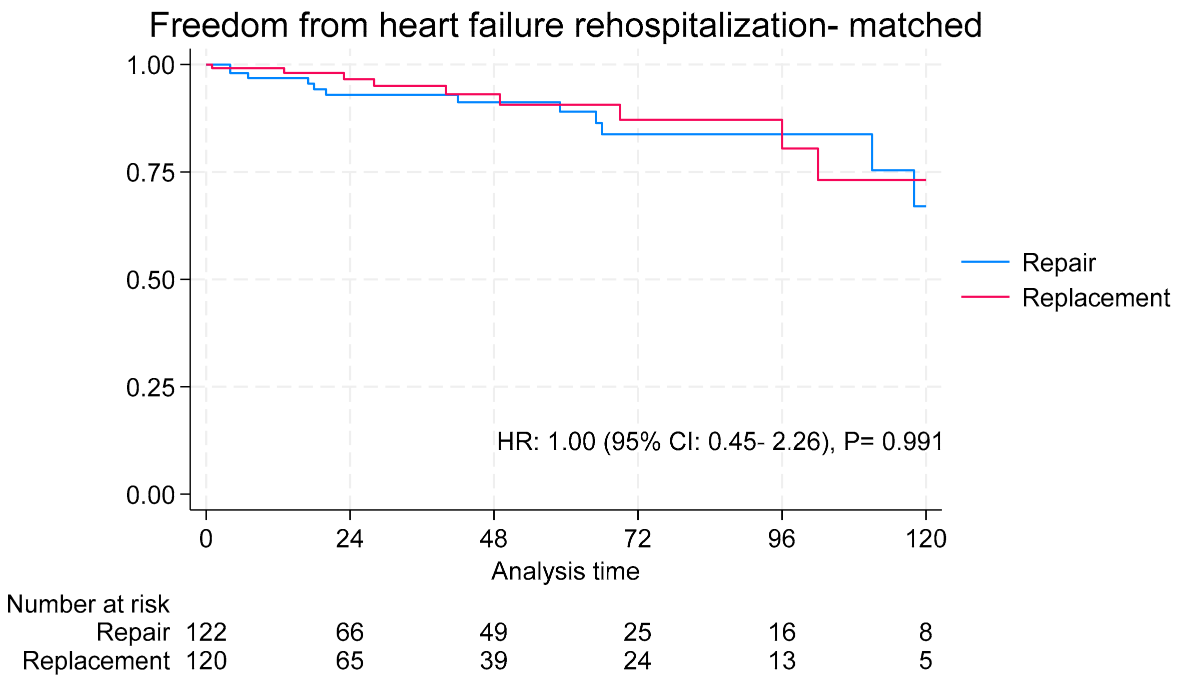

Figure S2: Freedom from heart failure rehospitalization in the unmatched (A) and unmatched (B) groups

A

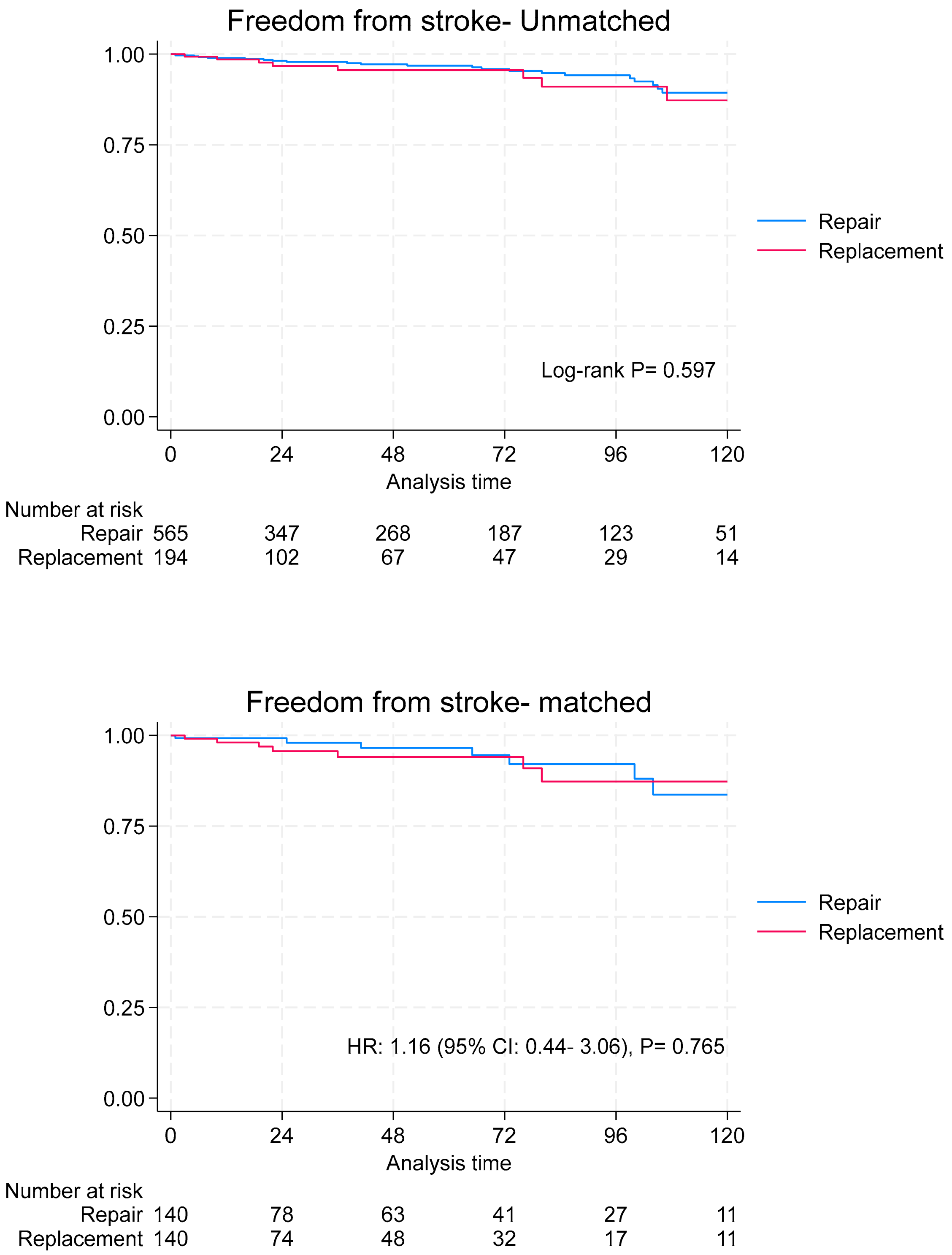

Figure S3: Freedom from stroke in the unmatched (A) and matched (B) groups

A

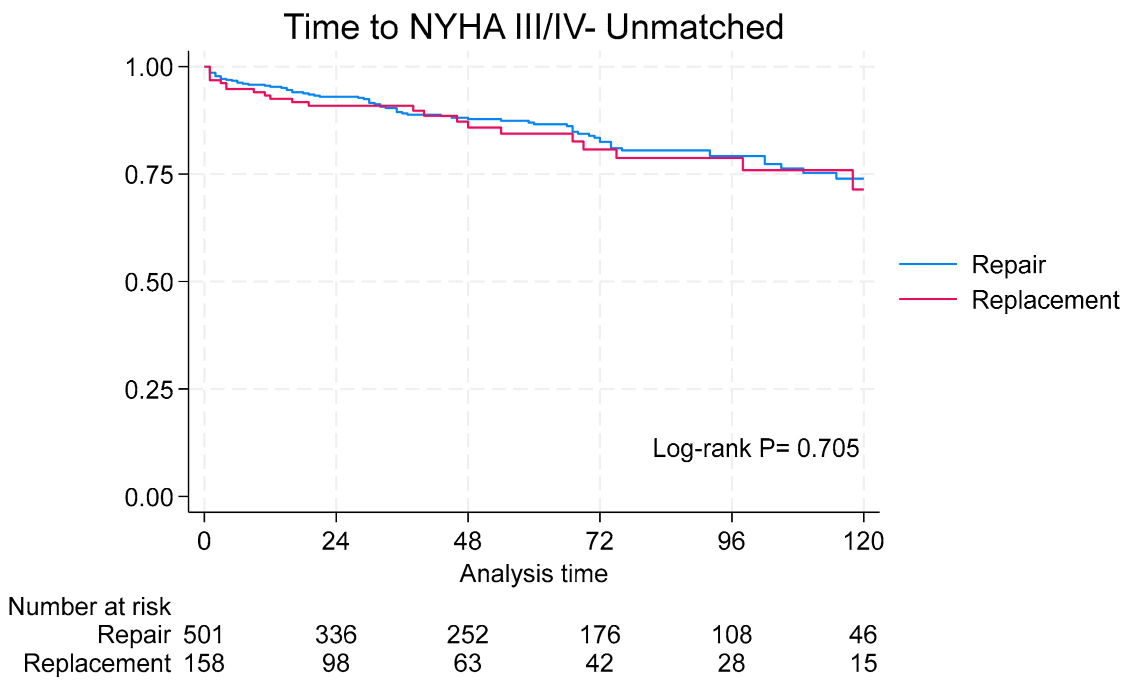

B

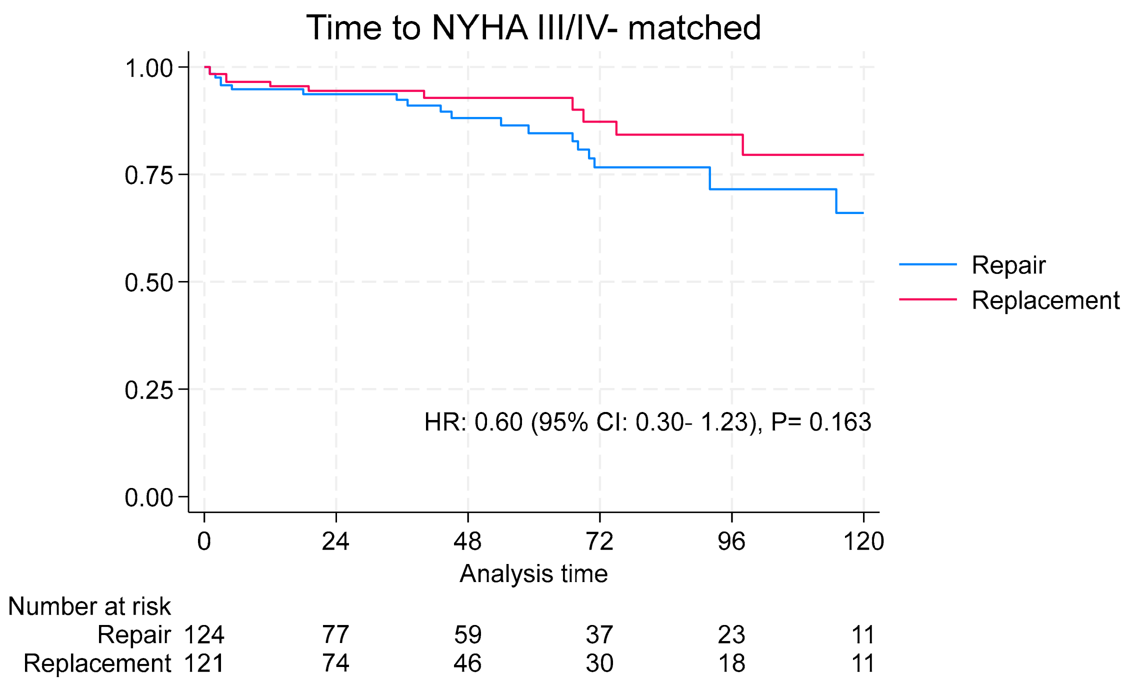

Figure S4: Freedom from NYHA class III/IV in the unmatched (A) and matched (B) groups
